# Enigma of COVID-19: is “multisystem inflammatory syndrome in adults” (MIS-A) predictable?

**DOI:** 10.1186/s12879-022-07303-8

**Published:** 2022-03-28

**Authors:** Istemi Serin, Nagehan Didem Sari, Murat Gunaltili, Ayse Karakilic, Begum Gulesir, Beyza Kal Kolik, Gulnihal Cevik, Hilal Sungurlu, Melike Keskin, Muhammed Baltik, Onurhan Cakmak, Tahir Alper Cinli

**Affiliations:** 1 Department of Hematology, Istanbul Training and Research Hospital, University of Health Sciences, Org.Nafiz GURMAN Cad. Fatih, 34098 Istanbul, Turkey; 2 Department of Clinical Microbiology and Infectious Diseases, Istanbul Training and Research Hospital, University of Health Sciences, Istanbul, Turkey; 3 Department of Internal Medicine, Istanbul Training and Research Hospital, University of Health Sciences, Istanbul, Turkey

**Keywords:** COVID-19, Multisystem inflammatory syndrome in adults (MIS-a), Mortality, Prognosis

## Abstract

**Background:**

Severe inflammation and one or more extrapulmonary organ dysfunctions have been reported and this clinical picture is defined as "multisystem inflammatory syndrome in adults" (MIS-A) in severe coronavirus disease-2019 (COVID-19). We aimed to determine the effect of LDH/lymphocyte ratio (LLR) on the development of MIS-A.

**Methods:**

The data of 2333 patients were retrospectively analyzed.

**Results:**

MIS-A rate was found to be 9.9% and MIS-A related mortality was 35.3%. LRR level above 0.24 was found to predict MIS-A development with 70% sensitivity and 65.2% specificity. The risk of MIS-A development was found to be 3.64 times higher in those with LRR levels above 0.24 compared to those with 0.24 and below. In patients with MIS-A, LRR level above 0.32 predicts mortality with 78% sensitivity and 70% specificity.

**Conclusions:**

Early detection of MIS-A with high sensitivity and specificity in a practical ratio is very important in terms new studies.

## Background

Coronavirus disease-2019 (COVID-19) is a disease caused by severe acute respiratory syndrome coronavirus-2 (SARS CoV-2), first detected in Wuhan, China in December 2019, and then affected the whole world and caused a pandemic [1]. As of March 21, 2021, more than 122 million people worldwide have been diagnosed with COVID-19, and the disease has killed 2.7 million people [2]. While the mortality rate of patients is approximately 2.5%, it increases to 16.6% in critically ill patients [3]. The group with chronic disorders such as hypertension, renal failure, chronic pulmonary disorders, diabetes or malignancy is most severely affected by the disease [4].

Approximately half of the patients do not have any significant symptoms, while most of them have symptoms such as fever, generalized muscle pain, cough and shortness of breath. However, in patients with severe pneumonia, severe morbidity and mortality, acute respiratory distress syndrome (ARDS), pulmonary edema, or multiple organ failure (MOF), can also be seen [5, 6]. Sudden ARDS, septic shock or MOF may also develop in patients with mild symptoms [7].

COVID-19 associated hyperinflammation overlaps with macrophage activation syndrome (MAS) in several ways in some patients. It has been shown that patients with severe COVID-19 pneumonia have increased cytokines similar to those in MAS [8]. Not only hypercytokinemia but also high serum ferritin, C-reactive protein (CRP) and D-dimer levels suggest the development of MAS-like severe inflammation in severe COVID-19 pneumonia [9]. However, the high levels of ferritin seen in COVID-19 are often not as high as expected in MAS. In addition, hepatosplenomegaly, low platelet and hypofibrinogenemia seen in MAS are not frequently seen in COVID-19-associated MAS-like pictures [10, 11]. Due to the MAS-like picture seen in COVID-19, researching the effectiveness of anti-cytokine treatments used in MAS in severe COVID-19 patients has become an important focus of interest. Various studies suggest that anti-cytokine therapies can be effective in patients with severe COVID-19 [12, 13].

Due to the reasons explained, the MAS-like picture seen in COVID-19 does not fully meet the classic MAS / hemophagocytic lymphohistiocytosis (HLH) criteria. Apart from the MAS-like picture, in various studies, in patients with acute or recent evidence of SARS-COV-2 infection, severe inflammation (e.g. CRP, ferritin, D-dimer, elevated cardiac and liver enzymes) and one or more extrapulmonary organ dysfunctions have been reported and this clinical picture is defined as "multisystem inflammatory syndrome in adults" (MIS-A) [14].

Before the definition of MIS-A, it is necessary to underline “multisystem inflammatory syndrome in children (MIS-C)”, which sheds light on MIS-A. The case definition for MIS-C is as follows: “An individual aged < 21 years presenting with fever, laboratory evidence of inflammation, evidence of clinically severe illness requiring hospitalization, with multisystem (> 2) organ involvement (cardiac, renal, respiratory, hematologic, gastrointestinal, dermatologic or neurologic); no alternative plausible diagnoses; positive for current or recent SARS-CoV-2 infection by PCR, serology, or antigen test or exposure to a suspected or confirmed COVID-19 case within the 4 weeks prior to the onset of symptoms” [15].

CRP, lactate dehydrogenase (LDH), increased ferritin levels, lymphopenia, and leukopenia are common laboratory findings in COVID-19 patients [16]. There are studies showing that factors such as advanced age, the presence of concomitant chronic diseases, lymphopenia, and high LDH levels are associated with increased mortality rates in COVID-19 patients [17, 18]. In COVID-19, organ and tissue damage may develop due to excessive inflammation and uncontrolled immune activation [19]. Therefore, inflammatory parameters such as neutrophils and CRP, cellular enzymes such as LDH and creatine kinase (CK) may be biomarkers that can be used to predict the prognosis of the disease. In COVID-19 patients, it is very important to identify potentially critical patients early, to predict the development of MIS-A and to initiate effective treatment in time. However, studies on biomarkers that can predict the prognosis of patients and the development of a MIS-A picture are limited.

A previous study at our center has shown that the LDH / lymphocyte ratio (LLR) can be used in the early diagnosis of COVID-19 and predicting mortality [20]. Based on this, we aimed to determine the effect of LLR on the development of MIS-A, early detection of critical patients and mortality in COVID-19 and MIS-A.

## Methods

Our study was conducted upon obtaining the approval from the local ethics committee and the relevant departments of the Ministry of Health. (Ethics committee approval number: 2689/ 22.01.2021, Ministry of Health Approval Number: 2021-01-07. T19.02.49.). The data of 2333 patients who were admitted to our hospital between March and December 2020; diagnosed as COVID-19 patients and hospitalized were retrospectively analyzed. Demographic data of patients such as age, gender, comorbidities such as chronic obstructive pulmonary disease (COPD), asthma, hypertension (HT), chronic renal failure (CRF), diabetes mellitus (DM), coronary artery disease (CAD) and malignancy, initial CT results (while examining CT findings, COVID-19 Reporting and Data System (CO-RADS) definitions were used and typical CT findings were presented [21]), initial leukocyte, neutrophil, lymphocyte, hemoglobin, platelet, urea, creatinine, D-Dimer, aspartate aminotransferase (AST), alanine transaminase (ALT), total protein, albumin, CK, CRP, procalcitonin, ferritin, fibrinogen, LDH, LLR values and their last status (alive or exitus) were recorded. 0–5 mg/dl for CRP; 30–400 mcg/L for ferritin were used as normal ranges.

The relationship between the patients' recorded data and individual survival was examined. In addition, the presence of resistant fever (presence of fever that continued for 3 days during the hospitalization period), the time (days) from the onset of symptoms to MIS-A and follow-up durations were also recorded.

### MIS-A definition

MIS-A case definition used in this study included the following five criteria [14]:

1. A severe illness requiring hospitalization in an individual aged ≥ 21 years;
2. Current or past infection with SARS-CoV-2: A positive test result for current or previous SARS-CoV-2 infection (nucleic acid, antigen, or antibody) during admission or in the previous 12 weeks;
3. Severe dysfunction in one or more extrapulmonary organ systems: Hypotension or shock, cardiac dysfunction, arterial or venous thrombosis or thromboembolism, or acute liver injury;
4. Laboratory evidence of elevated inflammatory markers: CRP, ferritin, D-dimer, interleukin [IL]-6);
5. Absence of severe respiratory illness: To exclude patients in which inflammation and organ dysfunction might be attributable simply to tissue hypoxia;
6. Absence of an alternative unifying diagnosis

### Exclusion criteria

Patients were excluded if alternative diagnoses such as bacterial sepsis were identified. In addition, patients with malignancy and similar comorbidities that may affect LLR were also excluded from the study.

Patients with severe respiratory symptoms other than mild respiratory symptoms, requiring invasive (intubation) or non-invasive respiratory support; in accordance with the MIS-A definition criteria, were excluded from the study.

While examining the laboratory results, its consistency was confirmed by looking at at least “2” test results. Cases who died in a short time or whose two recent results could not be obtained were excluded from the study.

Patients were excluded if alternative diagnoses such as bacterial sepsis were identified. To rule out bacterial sepsis, we looked for at least 2 negative hemocultures, Cases with all kinds of secondary conditions that may cause “tissue hypoxia” (severe metastatic malignancy, severe anemia, bleeding, etc.) were grouped as the excluded patients.

### Risk factors and sub-modeling

In order to show the effects of risk factors on mortality and MIS-A, four different model subgroups were created:

- Model I: Factors predicting MIS-A development in the whole population
- Model II: Factors predicting MIS-A development in surviving patients
- Model III: Factors predicting mortality in the whole population
- Model IV: Factors predicting mortality in MIS-A patients

### Statistical analysis

Statistical evaluation was performed using the Statistical Package for Social Sciences (SPSS) for Windows 20 (IBM SPSS Inc., Chicago, IL) program. The normal distribution of the data was evaluated with the Kolmogorov–Smirnov test. Normally distributed numerical variables were shown as mean ± standard deviation, and numerical variables not showing normal distribution shown as median (min–max). Categorical variables were expressed as numbers and percentages. Univariable Cox regression analysis was used to determine possible demographic and clinical risk factors associated with MIS-A and mortality. Independent predictors were determined by multivariate Cox regression analysis, which included risk factors found to be significant for each model (Model I-IV). The estimation value of LDH / Lymphocyte ratio to predict MIS-A and mortality was determined according to the Youden index method in ROC Curve analysis. Survival plots were shown by Kaplan–Meier analysis. P < 0.05 (*) value was considered significant in statistical analysis.

## Results

The study population consisted of 2333 patients (mean age: 59.4 ± 16.4, range: 21–101 years), including 1120 women and 1213 men. DM in 28.4% (n: 662), HT in 41.6% (n: 970), CRF in 8.4% (n: 196), CAD in 18.4% (n: 430), COPD in 7.5% (n: 174) and malignancy in 8.4% (n: 197) patients were found. In the whole population, the prevalence of MIS-A was 9.9% (n: 232).

Initial demographic and clinical findings associated with MIS-A in the whole cohort: Male gender, presence of typical COVID-19 pulmonary involvement in initial CT, low platelet, high neutrophil, low lymphocyte, high LDH, high LLR, high CRP, high urea, high AST (HR: 1.03; p = 0.006), high CK, high ferritin, high fibrinogen levels and presence of resistant fever (p < 0.05, for all) (Table 1).

**Table 1:** Initial demographic and clinical features associated with MIS-A development

	All patients n = 2333	MIS-A		Univariable Cox regression		
		No n = 2101	Yes n = 232	HR	95% CI	p
Age, years	59.4 ± 16.4	59.1 ± 16.6	62.1 ± 14.3	1.01	0.98–1.02	0.078
Gender, n (%)						
Female	1120 (48.0)	1031 (49.1)	89 (38.4)	Ref		
Male	1213 (52.0)	1070 (50.9)	143 (61.6)	1.53	1.18–2.00	0.002
Comorbidity, n (%)						
DM	662 (28.4)	591 (28.1)	71 (30.6)	1.06	0.80–1.40	0.674
HT	970 (41.6)	873 (41.6)	97 (41.8)	0.97	0.74–1.25	0.790
CRF	196 (8.4)	173 (8.2)	23 (9.9)	1.09	0.71–1.69	0.683
CAD	430 (18.4)	385 (18.3)	45 (19.4)	1.03	0.75–1.43	0.840
COPD	174 (7.5)	162 (7.7)	12 (5.2)	0.61	0.34–1.10	0.103
Malignancy	197 (8.4)	179 (8.5)	18 (7.8)	0.86	0.53–1.39	0.536
Presence of typical COVID-19 Involvement	1899 (81.4)	1681 (80.0)	218 (94.0)	3.67	2.14–6.30	< 0.001
Laboratory findings						
Hemoglobin (gr/dL)	12.6 ± 2.1	12.6 ± 2.1	12.7 ± 1.9	1.04	0.98–1.10	0.241
Platelet, × 10^3/^mm^3^	213 (1–1555)	216 (1–1555)	186.5 (24–685)	0.97	0.96–0.98	< 0.001
Leukocyte, × 10^3/^mm^3^	6.5 (0.1–124.4)	6.6 (0.1–124.4)	6.2 (0.6–40.4)	1.01	0.98–1.03	0.691
Neutrophil, × 10^3/^mm^3^	4.3 (0–42.4)	4.3 (0–42.4)	4.6 (0.2–35.8)	1.04	1.01–1.07	0.009
Lymphocyte, × 10^3^	1.3 (0.2–17.9)	1.3 (0.2–17.9)	1.0 (0.2–6.3)	0.46	0.35–0.58	< 0.001
LDH (IU/L)	251 (37–1877)	244 (37–1877)	331 (134–1323)	1.03	1.01–1.05	< 0.001
LLR	0.2 (0–4.2)	0.2 (0–0.7)	0.3 (0.1–4.2)	3.25	2.71–3.89	< 0.001
CRP (mg/dL)	39 (0.2–429)	34 (0.2–400)	77 (2.8–429)	1.06	1.04–1.07	< 0.001
Procalcitonin (ng/ml)	0.1 (0–81.7)	0.1 (0–81.7)	0.1 (0–27.9)	1.01	0.98–1.03	0.673
Urea (mg/dL)	33 (6–442)	32 (6–442)	40 (11–269)	1.04	1.01–1.07	0.003
Creatinine (mg/dL)	0.8 (0.1–29.4)	0.8 (0.1–29.4)	0.9 (0.4–17.9)	1.03	0.96–1.11	0.446
AST (IU/L)	26 (3–2524)	26 (3–2524)	33 (12–1251)	1.03	1.01–1.06	0.006
ALT (IU/L)	21 (1–2034)	20 (1–2034)	25 (5–631)	1.01	0.99–1.02	0.162
Total protein (gr/dL)	68.3 ± 7.3	68.3 ± 7.4	68 ± 6.5	0.99	0.98–1.02	0.679
Albumin (gr/L)	36.5 ± 5.2	36.6 ± 5.2	35.7 ± 5.2	0.98	0.95–1.01	0.075
CK (IU/L)	76 (1–7290)	73 (1–7290)	106.5 (12–3303)	1.03	1.01–1.06	0.003
D-dimer	0.7 (0–36)	0.7 (0–36)	0.8 (0–21.6)	0.99	0.95–1.04	0.876
Ferritin (mcg/L)	158 (2–5900)	145 (2–5900)	372 (14–3247)	1.08	1.07–1.09	< 0.001
Fibrinojen (mg/dL)	478.5 (0.7–12,000)	467 (0.7–12,000)	555 (139–1355)	1.02	1.01–1.04	0.002
Presence of Resistant Fever, n (%)	119 (5.1)	55 (2.6)	64 (27.6)	7.37	5.51–9.86	< 0.001
Time to blood sampling, days (range)	8 (1–22)	9 (4–22)	5 (1–12)	–	–	–
Time to MIS-A, days (range)	–	–	3 (1–26)	–	–	–
Exitus, n (%)	164 (7.0)	82 (3.9)	82 (35.3)	–	–	–
Follow up duration, days	7 (1–54)	6 (1–52)	10 (1–54)	–	–	–

Numerical variables were shown as mean ± standard deviation or median (min–max)

Categorical variables were shown as number (%)

p < 0.05 shows statistical significance

*MIS-A* multisystem inflammatory in adults, *HR* hazard ratio, *CI* confidence interval, *CT* computed tomography, *DM* diabetes mellitus, *HT* hypertension, *CRF* chronic renal failure, *CAD* coronary artery disease, *COPD* chronic obstructive pulmonary disease, *LDH* lactate dehydrogenase, *LLR* LDH / lymphocyte ratio, *CRP* C-reactive protein, *AST* aspartate aminotransferase, *ALT* alanine transaminase, *CK* creatine kinase

Initial demographic and clinical findings associated with mortality in the whole cohort: Advanced age, presence of typical COVID-19 pulmonary involvement in initial CT, low platelet, high leukocyte, high neutrophil, low lymphocyte, high LDH, high LLR, high CRP, high urea, low albumin, high CK, high ferritin, high fibrinogen levels, presence of MIS-A and time to MIS-A (p < 0.05, for all) (Table 2).

**Table 2:** Initial demographic and clinical features associated with mortality

	Survival		Univariable Cox Regression		
	Alive n = 2169	Exitus n = 164	HR	95% CI	p
Age, years	58.8 ± 16.4	67.9 ± 14.5	1.03	1.02–1.04	< 0.001
Gender, n (%)					
Female	1041 (48.0)	79 (48.2)			
Male	1128 (52.0)	85 (51.8)	1.01	0.74–1.38	0.937
Comorbidity, n (%)					
DM	603 (27.8)	59 (36.0)	1.34	0.97–1.84	0.074
HT	897 (41.4)	73 (44.5)	1.09	0.8–1.48	0.598
CRF	172 (7.9)	24 (14.6)	1.22	0.79–1.9	0.368
CAD	389 (17.9)	41 (25.0)	1.29	0.91–1.85	0.155
COPD	162 (7.5)	12 (7.3)	0.77	0.43–1.39	0.388
Malignancy	172 (7.9)	25 (15.2)	1.43	0.93–2.21	0.107
Presence of typical COVID-19 Involvement	1751 (80.7)	148 (90.2)	1.92	1.15–3.22	0.013
Laboratory findings					
Hemoglobin (gr/dL)	12.6 ± 2.1	12 ± 2.1	0.97	0.9–1.04	0.351
Plateler, × 10^3/^mm^3^	215 (1–1555)	190.5 (1.1–667)	0.98	0.97–0.99	0.006
Leukocyte, × 10^3/^mm^3^	6.5 (0.1–124.4)	7 (0.6–37.6)	1.02	1.01–1.04	0.021
Neutrophil, × 10^3/^mm^3^	4.2 (0–42.4)	5.7 (0.2–35.8)	1.07	1.04–1.10	< 0.001
Lymphocyte, × 10^3^	1.3 (0.2–17.9)	0.9 (0.2–2.1)	0.23	0.16–0.34	< 0.001
LDH (IU/L)	246.6 (37–1877)	341.5 (104–1293)	1.02	1.01–1.03	< 0.001
LLR	0.2 (0–0.7)	0.4 (0.1–4.2)	2.67	2.19–3.25	< 0.001
CRP (mg/dL)	35 (0.2–400)	95 (2.8–429)	1.06	1.04–1.08	< 0.001
Procalcitonin (ng/ml)	0.1 (0–81.7)	0.2 (0–53.3)	1.02	0.98–1.04	0.084
Urea (mg/dL)	32 (6–442)	47 (14–229.4)	1.06	1.03–1.09	< 0.001
Creatinine (mg/dL)	0.8 (0.1–29.4)	1 (0.4–7.3)	1.00	0.91–1.09	0.923
AST (IU/L)	26 (3–2524)	31 (11.4–229)	1.00	0.97–1.05	0.544
ALT (IU/L)	21 (1–2034)	22 (1–176)	1.00	0.99–1.01	0.245
Total protein (gr/dL)	68.3 ± 7.2	67.2 ± 8.1	0.99	0.97–1.01	0.228
Albumin (gr/L)	36.7 ± 5.1	33.9 ± 6.2	0.95	0.93–0.98	< 0.001
CK (IU/L)	74 (1–7290)	115.5 (17–3303)	1.04	1.02–1.07	0.001
D-dimer	0.7 (0–36)	1 (0.1–35.4)	1.04	1.01–1.07	0.019
Ferritin (mcg/L)	151.2 (2–5900)	327 (7.4–3247)	1.07	1.05–1.10	< 0.001
Fibrinojen (mg/dL)	472 (0.7–12,000)	602 (170–1355)	1.02	1.01–1.04	0.011
Presence of Resistant Fever, n (%)	94 (4.3)	25 (15.2)	1.15	0.73–1.81	0.549
MIS-A, n (%)	150 (6.9)	82 (50.0)	4.27	3.08–5.92	< 0.001
Time to blood sampling, days (range)	10 (4–22)	4 (1–12)	1.06	1.01–1.05	< 0.001
Time to MIS-A, days (range)	4 (1–26)	3 (1–20)	0.87	0.84–0.90	< 0.001
Follow up duration, days	6 (1–52)	7 (1–54)	–	–	–

Numerical variables were shown as mean ± standard deviation or median (min–max)

Categorical variables were shown as number (%)

p < 0.05 shows statistical significance

*MIS-A* multisystem inflammatory in adults, *HR* hazard ratio, *CI* confidence interval, *CT* computed tomography, *DM* diabetes mellitus, *HT* hypertension, *CRF* chronic renal failure, *CAD* coronary artery disease, *COPD* chronic obstructive pulmonary disease, *LDH* lactate dehydrogenase, *LLR* LDH / lymphocyte ratio, *CRP* C-reactive protein, *AST* aspartate aminotransferase, *ALT* alanine transaminase, *CK* creatine kinase

Initial demographic and clinical findings associated with MIS-A, in the subgroup analysis where patients with a mortal course were excluded: Male gender, presence of typical COVID-19 pulmonary involvement in initial CT, low platelet, low lymphocyte, high LDH, high LLR, high CRP, high AST and high ferritin levels and presence of resistant fever (p < 0.05, for all) (Table 3).

**Table 3:** MIS-A risk compared to non-MIS-A and alive patients; mortality in MIS-A

	MIS-A (−) & Alive n = 2019	MIS-A ( +)		Univariable Cox Regression^a^			Univariable Cox Regression^b^		
		Alive n = 150	Exitus n = 82	HR	95% CI	p	HR	95% CI	p
Age, years	58.7 ± 16.5	59.4 ± 14	67 ± 13.7	1.00	0.99–1.01	0.878	1.03	1.01–1.05	< 0.001
Gender, n (%)									
Female	990 (49.0)	51 (34.0)	38 (46.3)	Ref					
Male	1029 (51.0)	99 (66.0)	44 (53.7)	1.83	1.31–2.57	< 0.001	0.82	0.53–1.27	0.365
Comorbidity, n (%)									
DM	560 (27.7)	43 (28.7)	28 (34.1)	0.97	0.68–1.38	0.866	1.39	0.87–2.21	0.164
HT	834 (41.3)	63 (42.0)	34 (41.5)	0.97	0.70–1.34	0.832	1.21	0.77–1.90	0.415
CRF	158 (7.8)	14 (9.3)	9 (11.0)	1.06	0.61–1.84	0.841	1.20	0.60–2.41	0.599
CAD	360 (17.8)	29 (19.3)	16 (19.5)	1.05	0.70–1.58	0.796	0.87	0.50–1.51	0.620
COPD	154 (7.6)	8 (5.3)	4 (4.9)	0.63	0.31–1.28	0.202	0.90	0.33–2.48	0.845
Malignancy	164 (8.1)	8 (5.3)	10 (12.2)	0.61	0.30–1.24	0.171	1.22	0.62–2.40	0.565
Presence of typical COVID-19 Involvement	1609 (79.7)	142 (94.7)	76 (92.7)	4.33	2.12–8.83	< 0.001	0.84	0.37–1.94	0.685
Laboratory findings									
Hemoglobin (gr/dL)	12.8 ± 2.1	13.0 ± 1.6	12.1 ± 2.3	1.08	0.98–1.21	0.205	0.91	0.81–1.01	0.088
Platelet, × 10^3/^mm^3^	217 (1–1555)	187 (24–685)	182 (25–409)	0.97	0.96–0.99	0.008	0.99	0.96–1.02	0.407
Leukocyte, × 10^3/^mm^3^	6.5 (0.1–124.4)	5.9 (1.2–40.4)	6.8 (0.6–37.6)	0.97	0.92–1.01	0.133	1.05	1.02–1.08	0.003
Neutrophil, × 10^3/^mm^3^	4.3 (0–42.4)	4 (1.1–30.7)	5.4 (0.2–35.8)	0.99	0.95–1.04	0.718	1.06	1.03–1.10	0.001
Lymphocyte, × 10^3^	1.4 (0.2–17.9)	1.2 (0.4–6.3)	0.8 (0.2–2.1)	0.67	0.51–0.87	0.003	0.25	0.13–0.45	< 0.001
LDH (IU/L)	243 (37–1877)	307.5 (134–1323)	379 (174–1293)	1.02	1.01–1.03	< 0.001	1.02	1.01–1.03	< 0.001
LLR	0.2 (0–0.7)	0.3 (0.1–0.6)	0.5 (0.2–4.2)	9.41	3.62–24.45	< 0.001	1.90	1.49–2.41	< 0.001
CRP (mg/dL)	32 (0.2–400)	63.3 (3.6–310)	111.4 (2.8–429)	1.04	1.02–1.06	< 0.001	1.05	1.02–1.07	< 0.001
Procalcitonin (ng/ml)	0.1 (0–81.7)	0.1 (0–5.3)	0.3 (0–27.9)	0.94	0.82–1.07	0.330	1.01	0.97–1.06	0.574
Urea (mg/dL)	32 (6–442)	37.9 (11–269)	49.1 (19–211)	1.00	0.99–1.01	0.557	1.05	1.01–1.09	0.017
Creatinine (mg/dL)	0.8 (0.1–29.4)	0.9 (0.4–17.9)	0.9 (0.5–7.3)	1.02	0.92–1.12	0.697	0.98	0.88–1.10	0.778
AST (IU/L)	26 (3–2524)	31.8 (12–1251)	34 (12–229)	1.03	1.01–1.05	0.026	1.00	0.99–1.02	0.930
ALT (IU/L)	20 (1–2034)	25 (5–631)	23.3 (7–176)	1.00	0.98–1.04	0.125	1.00	0.99–1.00	0.660
Total protein (gr/dL)	68.3 ± 7.3	68.5 ± 6.4	67.0 ± 6.6	1.00	0.98–1.03	0.796	0.97	0.93–1.01	0.116
Albumin (gr/L)	36.7 ± 5.1	36.7 ± 4.5	33.7 ± 5.7	1.01	0.98–1.05	0.403	0.95	0.91–0.98	0.003
CK (IU/L)	72 (1–7290)	103 (12–1151)	127.5 (17–3303)	1.03	0.98–1.06	0.087	1.08	1.01–1.16	0.020
D-dimer	0.7 (0–36)	0.7 (0–21.6)	1.1 (0.1–17)	0.98	0.91–1.05	0.535	1.04	0.97–1.12	0.291
Ferritin (mcg/L)	143 (2–5900)	346.5 (14–2000)	470 (18–3247)	1.08	1.06–1.10	< 0.001	1.05	1.01–1.09	0.009
Fibrinojen (mg/dL)	465 (0.7–12,000)	535 (139–1081)	614 (170–1355)	1.02	0.99–1.04	0.155	1.22	1.11–1.34	< 0.001
Presence of Resistant Fever, n (%)	49 (2.4)	45 (30.0)	19 (23.2)	9.04	6.34–12.89	< 0.001	0.50	0.29–0.87	0.013
Time to blood sampling, days (range)	10 (4–22)	6 (3–16)	3 (1–11)	–	–	–	–	–	–
Follow up duration, days	6 (1–52)	11 (4–42)	7 (1–54)	–	–	–	–	–	–

Numerical variables were shown as mean ± standard deviation or median (min–max)

Categorical variables were shown as number (%)

p < 0.05 shows statistical significance

*MIS-A* multisystem inflammatory in adults, *HR* hazard ratio, *CI* confidence interval, *CT* computed tomography, *DM* diabetes mellitus, *HT* hypertension, *CRF* chronic renal failure, *CAD* coronary artery disease, *COPD* chronic obstructive pulmonary disease, *LDH* lactate dehydrogenase, *LLR* LDH / lymphocyte ratio, *CRP* C-reactive protein, *AST* aspartate aminotransferase, *ALT* alanine transaminase, *CK* creatine kinase

^a^MIS-A risk in in surviving patients (Alive MIS-A vs non-MIS-A)

^b^Mortality risk in MIS-A patients (Exitus MIS-A vs Alive MIS-A)

Initial demographic and clinical findings associated with mortality in MIS-A patients compared to survivors: Advanced age, high leukocyte, high neutrophil, low lymphocyte, high LDH, high LLR, high CRP, high urea, low albumin, high CK, high ferritin and high fibrinogen levels (p < 0.05, for all) (Table 3).

Multivariate regression models that include MIS-A and possible risk factors associated with mortality are shown in Table 4. To determine the independent predictors of MIS-A, Model I was created for the entire cohort and Model II was created for the subgroup in which patients with a mortal course were excluded. According to this; in Model I, male gender, presence of typical COVID-19 pulmonary involvement in initial CT, low platelet, high LLR and high CRP level (p < 0.05, for all) were found to be independent predictors of MIS-A in the whole cohort. Similar independent predictors were found in Model II, except for platelet level (Table 4).

**Table 4:** Independent predictors of MIS-A and mortality

	HR	95% CI	p
*Model I*			
Gender			
Female	Ref		
Male	1.39	1.06–1.82	0.019
Presence of typical COVID-19 Involvement	3.37	1.96–5.79	< 0.001
Platelet	0.98	0.97–0.99	0.026
LLR	2.48	1.98–3.09	< 0.001
CRP	1.03	1.03–1.06	< 0.001
	− 2Log Likelihood = 3293.5; p < 0.001		
*Model II*			
Gender			
Female	Ref		
Male	1.65	1.17–2.32	0.004
Presence of typical COVID-19 Involvement	4.1	2.01–8.37	< 0.001
LLR	4.82	1.70–13.65	0.003
CRP	1.03	1.01–1.05	0.023
	− 2Log Likelihood = 3293.5; p < 0.001		
*Model III*			
Age	1.03	1.02–1.04	< 0.001
LLR	1.44	1.13–1.84	0.003
CRP	1.05	1.03–1.07	< 0.001
MIS-A	4.77	3.20–7.09	< 0.001
	− 2Log Likelihood = 1941.4; p < 0.001		
*Model IV*			
Age	1.03	1.01–1.04	0.003
LLR	1.37	1.05–1.80	0.023
CRP	1.04	1.02–1.07	0.001
	− 2Log Likelihood = 718.3; p < 0.001		

Model I: Factors predicting MIS-A development in the whole population

Model II: Factors predicting MIS-A development in surviving patients

Model III: Factors predicting mortality in the whole population

Model IV: Factors predicting mortality in MIS-A patients

*MIS-A* multisystem inflammatory in adults, *HR* hazard ratio, *CI* confidence interval, *LLR* LDH / lymphocyte ratio, *CRP* C-reactive protein

Model III for the entire cohort and Model IV for MIS-A subgroup were created to determine independent predictors of mortality. Advanced age, high LRR, high CRP and presence of MIS-A in Model III (p < 0.05, for all) were found to be independent predictors of mortality in the whole cohort. In Model IV, advanced age, high LRR and high CRP (p < 0.05, for all); they were found to be independent predictors of mortality in MIS-A patients (Table 4).

**Fig. 1:**
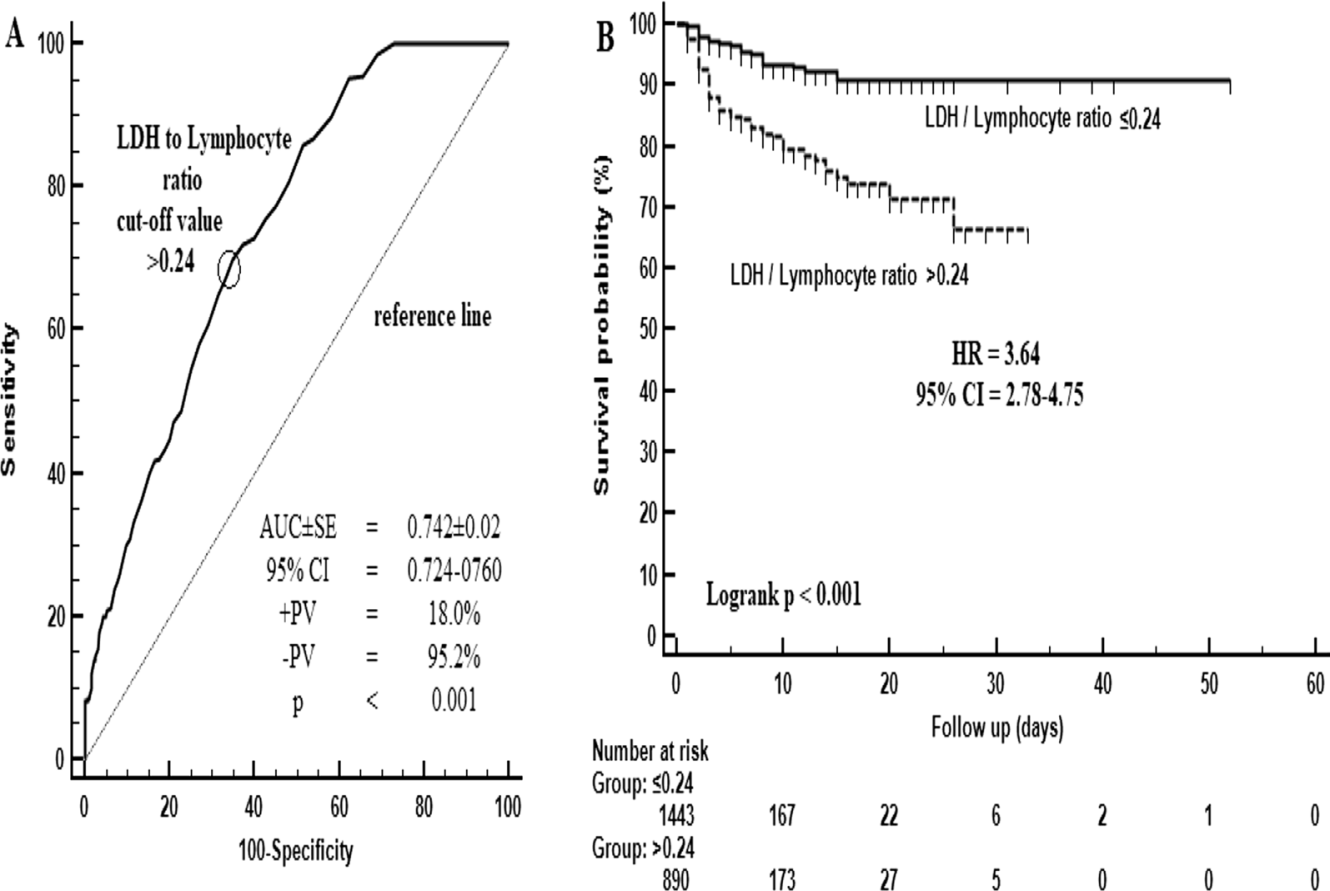
Diagnostic performance of the LDH / lymphocyte ratio in predicting MIS-A (**A**) and the risk factor according to the predictive value (**B**)

**Fig. 2:**
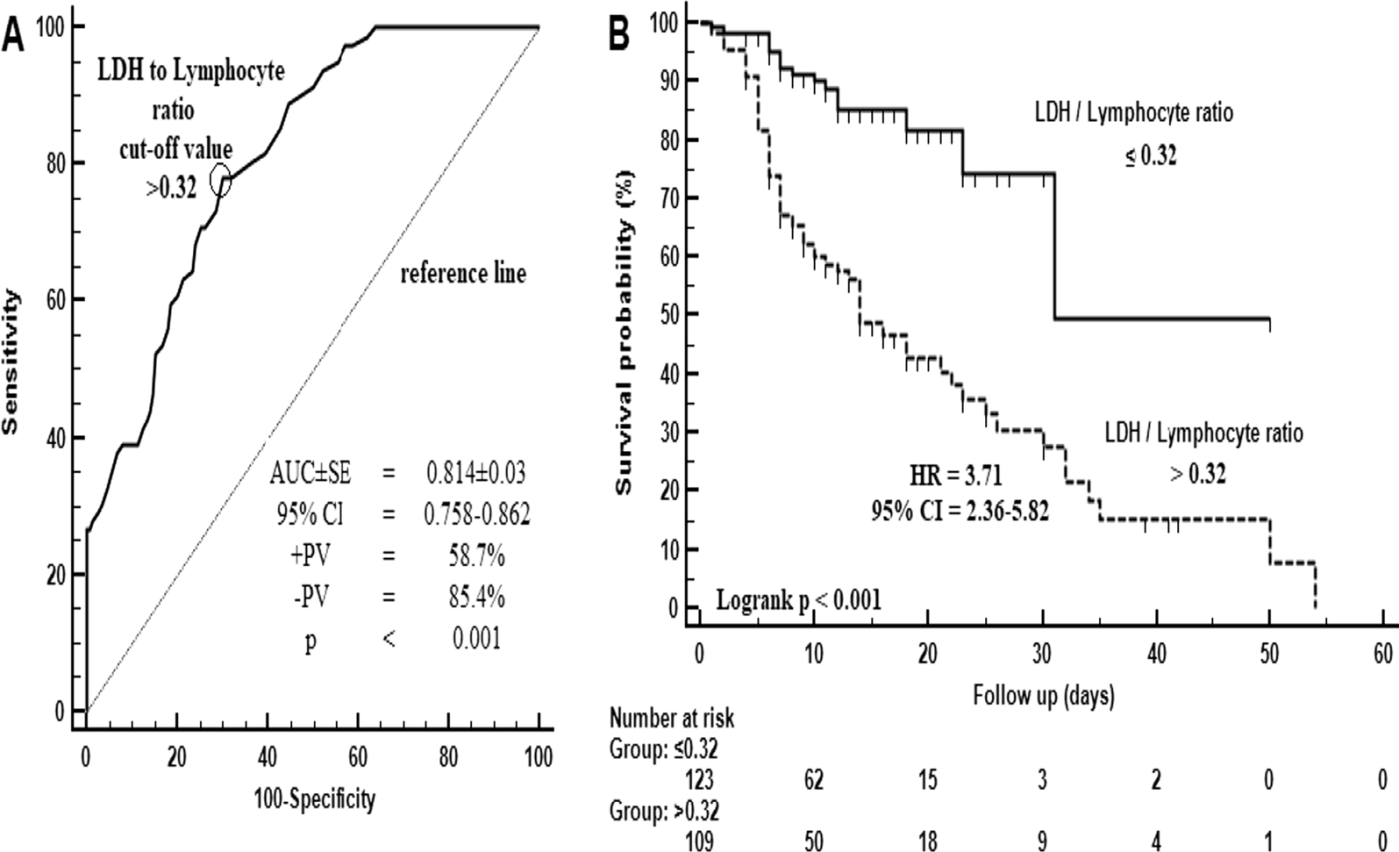
Diagnostic performance of LDH / lymphocyte ratio in predicting mortality (**A**) and risk factor according to predictive value (**B**) in MIS-A patients

In the whole cohort, LRR level above 0.24 was found to predict MIS-A development with 70% sensitivity and 65.2% specificity (AUC ± SE = 0.742 ± 0.02; + PV: 18%; -PV: 95.2%; p < 0.001) (Fig. 1A). The risk of MIS-A development was found to be 3.64 times higher (HR: 3.64; 95% CI = 2.78–4.75; p < 0.001) in those with LRR levels above 0.24 compared to those with 0.24 and below (Fig. 1B). In patients with MIS-A, it was determined that LRR level above 0.32 predicts mortality with 78% sensitivity and 70% specificity (AUC ± SE = 0.814 ± 0.03; + PV: 58.7%; -PV: 85.4%; p < 0.001) (Fig. 2A). In patients with MIS-A, the mortality risk was found to be 3.71 times higher (HR: 3.71; 95% CI = 2.36–5.82; p < 0.001) in patients with LRR level above 0.32 compared to those with a LRR level above 0.32 and below (HR: 3.71; 95% CI = 2.36–5.82) (Fig. 2B).

The predictive value of LRR for mortality in the whole cohort was 0.23 (AUC ± SE = 0.808 ± 0.02; Sensitivity 86%; Specificity: 62.7%; + PV = 14.8%; -PV = 98.3%; p < 0.001). Except for Model II, LLR was found to have superior diagnostic performance compared to LDH in other models.

## Discussion

In our study, we aimed to reveal the clinical features and mortality of MIS-A in our COVID-19 cases and effect of LLR on MIS-A prediction in a large patient group. Many studies have been conducted regarding the COVID-19 associated cytokine storm and the developing MAS-like clinical picture. It is possible to say that there are studies in which many new criteria have been developed and examined, since the clinical picture encountered does not comply with the previously reported MAS / HLH criteria. In order to see and examine the criteria from literature together, they are summarized in Table 5. For this purpose, COVID-19 associated cytokine storm or MIS-A that we have taken as basis in this study can be considered among the “cytokine storm syndromes” (CSS) [22]. These syndromes consist of different clinical pictures in which more than one etiology may play a role in the clinic.

**Table 5:** 2016 MAS, HLH-2004, HScore and MIS-A Criteria

2016 MAS Criteria [29]	HLH-2004 [30]	HScore [31]	MIS-A [14]
•Ferritin > 684 mcg/L and any 2 of following: •Platelet count 181 × 10^3^/mm^3^ •Triglycerides > 156 mg/dl •Fibrinogen ≤ 360 mg/dl •AST > 48 IU/L	The diagnosis HLH can be established if one of either 1 or 2 below is fulfilled: (1) A molecular diagnosis consistent with HLH (2) Diagnostic criteria for HLH fulfilled (five out of the eight criteria below) (A) Initial diagnostic criteria (to be evaluated in all patients with HLH) •Fever •Splenomegaly •Cytopenias (affecting 2 of 3 lineages in the peripheral blood): Hemoglobin < 9 g/dL (in infants < 4 weeks: hemoglobin < 10 g/dL) Platelets < 100 × 10^3/^mm^3^ Neutrophils < 1 × 10^3/^mm^3^ •Hypertriglyceridemia and/or hypofibrinogenemia: Fasting triglycerides ≥ 265 mg/dl •Fibrinogen ≤ 150 mg/dL •Hemophagocytosis in bone marrow or spleen or lymph nodes •No evidence of malignancy (B) New diagnostic criteria •Low or absent NK-cell activity (according to local laboratory reference) •Ferritin ≥ 500 mcg/L •Soluble CD25 ≥ 2,400 U/ml	•Known underlying immunosuppression: 0 points (no) or 18 (yes) •Temperature 0 (< 38.4), 33 (38.4–39.4), or 49 (> 39.4) •Organomegaly 0 (no), 23 (hepatomegaly or splenomegaly) or 38 (hepatomegaly and splenomegaly) •Cytopenia 0 (one lineage), 24 (two lineages) or 34 (three lineages) •Ferritin (mcg/L) 0 (< 2000), 35 (2000–6000), or 50 (> 6000) •Triglyceride (mg/dL) 0 (< 150), 44 (150–400), or 64 (> 400) •Fibrinogen (mg/dL) 0 (> 250) or 30 (≤ 250) •AST (IU/L) 0 (< 30) or 19 (≥ 30) •Hemophagocytosis features on bone marrow aspirate 0 (no) or 35 (yes) *The best cutoff value for HScore was 169, corresponding to a sensitivity of 93%, a specificity of 86%, and accurate classification of 90% of the patients	1. A severe illness requiring hospitalization in an individual aged ≥ 21 years; 2. Current or past infection with SARS-CoV-2; 3. Severe dysfunction in one or more extrapulmonary organ systems; 4. Laboratory evidence of elevated inflammatory markers (e.g., CRP, ferritin, D-dimer, interleukin [IL]-6); 5. Absence of severe respiratory illness; 6. Absence of an alternative unifying diagnosis

Other points of difference between MIS-A and other CSS encountered in the course of COVID-19 are also identified. Neutrophilia, high fibrinogen, low albumin and relatively normal triglyceride levels seen in COVID-19 associated cytokine storm (CS) and MIS-A; reveals the presence of a different clinical picture from other CSS [23–25].

In a study investigating “HScore” and COVID-19 related CS [26], the limitations of HScore are clearly revealed. Lymphopenic leukocytosis that develops during COVID-19 is defined as an important limitation point for HScore where leukopenia takes place. Although hyperferritinaemia, a hallmark of secondary HLH and HScore, ferritin concentrations rarely reach the HScore threshold of 2000 mcg / L until late in COVID-19. Other HScore criteria such as hypertriglyceridaemia, splenomegaly, hepatomegaly, and bone marrow hemophagocytosis are not reported in COVID-19. While high fever indicates higher scoring in HScore, this is not expected in the course of COVID-19. In our study, median leukocyte 6.2 × 10^3^ / mm^3^ (0.6–40.4) in the MIS-A group, ferritin 372 mcg / L (14–3247), and presence of resistant fever was 27,6% with 64 patients. In the light of all these data, it can be said that the COVID-19 MIS-A is quite different from the classical MAS / HLH.

In the study conducted by Caricchio et al. [27], we see that COVID-19 related CS and MAS 2016, HLH, HScore are compared. In this study, which showed that COVID-19 CS and other clinical definitions were not fully compatible, it was shown that the criterion system developed was effective in the diagnosis of COVID-19 CS with 0,85 sensitivity and 0,80 specificity. The criterion system discussed in this study included three main headings: (1) Inflammation, (2) Cell death and tissue damage, (3) Prerenal electrolyte imbalances. In our study, a LLR level of above 0.24 predicts MIS-A with 70% sensitivity and 65.2% specificity and above 0.32 predicted mortality with 78% sensitivity and 70% specificity. Providing a very high sensitivity and specificity alone is an important achievement in terms of the literature.

In the study of Webb et al. [28], a new criteriaization system has been developed to define COVID-19 associated hyperinflammatory syndrome (cHIS). For this purpose, it has been associated with cHIS with 6 separate criteria: Fever, macrophage activation (hyperferritinaemia), hematological dysfunction (neutrophil to lymphocyte ratio), hepatic injury (elevated LDH or AST), coagulopathy (elevated D-dimer), and cytokinaemia (elevated CRP, interleukin-6 or triglycerides). With this scale, hospitalization mortality and need for mechanical ventilation were examined. A total of 299 patients with COVID-19 diagnosed between March 13 and May 5, 2020, were included in this study. Unadjusted discrimination of the maximum daily cHIS score was 0.81 (95% CI 0.74–0.88) for in-hospital mortality and 0.92 (0.88–0.96) for mechanical ventilation; in multivariable analysis (odds ratio 1.6 [95% CI 1.2–2.1], p = 0.0020, for mortality and 4.3 [3.0–6.0], p < 0 0001, for mechanical ventilation). 161 (54%) of 299 patients met two or more cHIS criteria during their hospital admission; these patients had higher risk of mortality than patients with a score of less than 2 (24 [15%] of 138 vs one [1%] of 161) and for mechanical ventilation (73 [45%] vs three [2%]). In this study, similar clinical and laboratory parameters were used to examine the previous COVID-19-associated hyperinflammatory response, and more laboratory-related parameters were used when compared with the MIS-A criteria.

COVID-19 MIS-A constitutes an important area of study for the literature and there are many missing points. In our patient group, the MIS-A rate was 9.9%. Overall mortality was 7%, while it was 35.3% in the MIS-A group. Because of the significant mortality difference, the use of LLR as a predictive factor in MIS-A will be a clinically important step. The time between the onset of infection and the MIS-A is also quite uncertain. When the literature is reviewed, the median time to the onset of critical illness after the first symptom is 10–12 and to MIS-A is 2–5 days. In our study, MIS-A clinical picture developed in a median of 6 days (range: 1–52) [22, 23]. It should be emphasized that this period revealed in the literature was determined by the examination of a limited number of patients or case series. We achieved a more sensitive value of median time to MIS-A in a much larger population.

Our study had important limitations. Since the study was conducted only in inpatients, it can be said that the mortality was higher than expected. In addition, not checking an important MIS-A marker such as IL-6 in every patient may have kept the patient group narrower than expected.

## Conclusions

As a result, in our study, MIS-A rate was found to be 9.9% and MIS-A related mortality was 35.3%. In our study, LLR level of above 0.24 predicts MIS-A with 70% sensitivity and 65.2% specificity and above 0.32 predicted mortality with 78% sensitivity and 70% specificity. The high mortality rate of MIS-A in the course of COVID-19, and the detection of MIS-A with high sensitivity and specificity in a practical ratio is very important in terms of literature and new studies.

## Data Availability

The authors declare that data supporting the findings of this study are available within the referenced articles.

## Abbreviations

COVID-19: Corona virus disease-2019
Sars-Cov-2: Severe acute respiratory syndrome coronavirus-2
ARDS: Acute respiratory distress syndrome
MOF: Multiple organ failure
MAS: Macrophage activation syndrome
CRP: C-reactive protein
HLH: Hemophagocytic lymphohistiocytosis
MIS-A: Multisystem inflammatory syndrome in adults
LDH: Lactate dehydrogenase
CK: Creatine kinase
LLR: LDH / lymphocyte ratio
COPD: Chronic obstructive pulmonary disease
HT: Hypertension
CRF: Chronic renal failure
DM: Diabetes mellitus
CAD: Coronary artery disease
CO-RADS: COVID-19 Reporting and Data System
AST: Aspartate aminotransferase
ALT: Alanine transaminase
HR: Hazard ratio
MIS-C: Multisystem Inflammatory Syndrome in Children
CHIS: COVID-19 associated hyperinflammatory syndrome
